# Effect of Botulinum Toxin on Sensori–Motor Integration in Movement Disorders: A Scoping Review

**DOI:** 10.3390/toxins17080416

**Published:** 2025-08-16

**Authors:** Animesh Das, Mandar Jog

**Affiliations:** Department of Clinical Neurological Sciences, Western University, London, ON N6A 3K7, Canada; animeshdas05@gmail.com

**Keywords:** sensori-motor integration, botulinum toxin, basal ganglia

## Abstract

**Background:** The primary effect of Botulinum toxin (BoNT) is to cause weakness in the injected muscles by inhibiting the release of acetyl choline from presynaptic nerve terminals. Its effect on sensorimotor integration (SMI) has largely been confined to small studies. The aim of this review is to highlight effect of BoNT on SMI in the context of Parkinson’s disease (PD), Cervical dystonia (CD), and Writer’s cramp (WC). **Methods:** Using keywords “Botulinum toxin” and “sensorimotor integration” or “Freezing of gait (FOG)” or ‘Tremor”or “Cervical dystonia” or “Parkinson’s disease”, or “Writer’s cramp”, PubMed database was searched for relevant articles supporting our view. The abstracts of all resultant articles (case reports, case series, randomized trials, observational studies) were reviewed to look for evidence of effects of botulinum toxin on SMI. The relevant articles were charted in excel sheet for further full text review. **Results:** In FOG, chronic BoNT injections may alter central motor patterns with inclusion of alternative striatal systems, cerebellum, and its connections. In tremor, the afferent proprioceptive input may be modified with reduction of intracortical facilitation and increment of intracortical inhibition. In CD, BoNT can restore disorganized cortical somatotrophy, the key pathophysiology behind cervical dystonia. Similarly, in WC, both the deficient sensory system and abnormal reorganization of the sensorimotor cortex may be altered following chronic BoNT injections. **Conclusions:** There is preliminary evidence that BoNT may modulate SMI in PD, CD, and WC by altering inputs from the muscle spindles in short term and modifying circuits/particular anatomic cerebral cortices in the long term. Properly conducted randomized trials comparing BoNT with placebo or prospective large-scale studies to look for effect on various surrogate markers reflective of changes in SMI should be the next step to confirm these findings. Targeting the system of afferents like spindles and golgi tendon organs in muscles may be a better way of injecting BoNT, with lower amounts of toxin needed and potential for lesser side-effects like weakness and atrophy. However, this needs to be proven in controlled trials.

## 1. Introduction

To perform a voluntary movement, the integration of a normally functioning motor and sensory system is paramount. The motor system cannot execute a command without information from the sensory system about the condition of oneself in space. Multi-sensory integration is one of the parts of sensory–motor integration where the sensory system integrates the information it gathers through various modalities and provides an accurate perceptual estimate to the motor system. The motor system can then plan and modulate the output according to the demand of the environment. This whole process is called sensory–motor integration, or SMI [1]. With any deficit in the SMI, as expected, there will be difficulty in performing any motor movement.

A typical motor movement consists of ideation, planning, motivation, and execution. The prefrontal cortex is involved in ideation. Lateral premotor and medial supplementary motor cortices plan the movement. With the sensory inputs received through four main systems, namely somatosensory, visual, auditory, and vestibular systems, the parietal cortex also plays an active role in planning [2]. The limbic system is involved in motivation.

**Figure 1:**
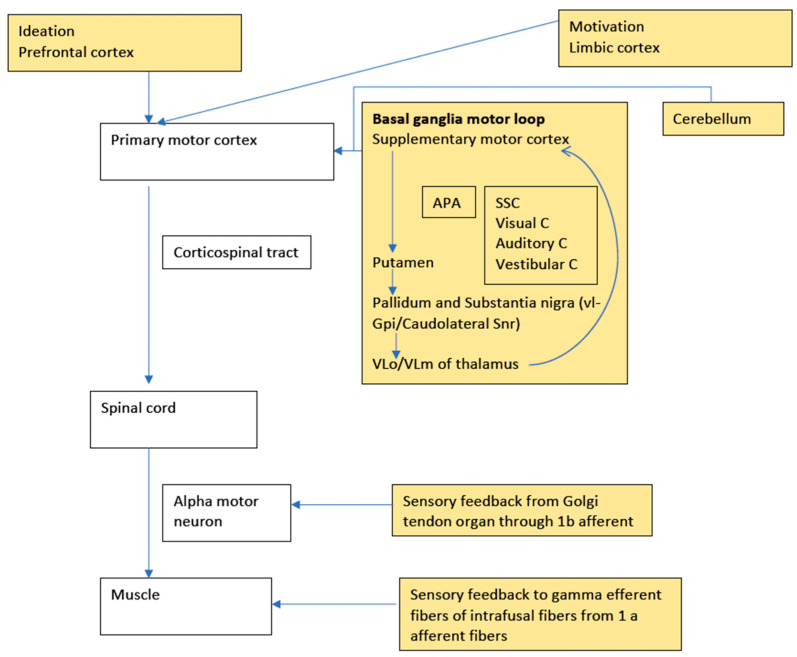
A box diagram showing human motor control with the concept of sensorimotor integration. The boxes in white depict the primary motor pathway, which is modulated by various inputs from the yellow boxes. A defect in any of these multiple levels can have issues with sensori–motor integration. APA: Arcuate Premotor cortex, SSC: Somatosensory cortex, Visual C: Visual cortex, Auditory C: Auditory cortex, Vestibular C: Vestibular cortex, vl-Gpi: Ventrolateral Globus pallidus interna, Caudolateral SNr: Caudolateral substantia nigra pars reticulata, VLo: ventralis lateralis pars oralis, VLm: ventralis lateralis pars medialis.

While planning a motor action, at least four circuits of the basal ganglia are involved, namely the motor loop related to learned movements, the cognitive loop with motor intentions, the limbic loop with the emotional aspects of the movement, and the oculomotor loop with voluntary saccades [2]. The most relevant (to this review) motor loop starts with the supplementary motor cortex (SMA) projecting to the putamen (with additional inputs from arcuate premotor area, motor cortex, and somatosensory cortex), which in turn projects to ventrolateral globus pallidus interna and caudolateral substantia nigra pars reticulata, which again projects to ventralis lateralis pars oralis and ventralis lateralis pars medialis nuclei of the thalamus. Finally, the output reaches the SMA to complete the loop [3]. A box figure of human motor control with the concept of sensori–motor integration is depicted in Figure 1.

With this background of SMI and how movement is initiated, this review would delve into the pathways through which botulinum toxin (BoNT) may act in conditions like Parkinson’s disease (PD), cervical dystonia (CD), and writer’s cramp (WC). These three conditions were selected as there is preliminary evidence to suggest that SMI is altered in these disorders.

When BoNT is injected peripherally, the N terminal of the heavy chain promotes penetration and translocation of the light chain across the endoplasmic membrane to cytosol [4]. The light chain then cleaves the fusion protein SNAP 25 (synaptosomal-associated protein), inhibiting the calcium-mediated release of acetylcholine from the presynaptic nerve terminal, thereby producing weakness of the injected muscles. BoNT type B, D, F, and G cleave VAMP (vesicular-associated membrane protein) and type C cleave syntaxin and SNAP25. SNAP 25, VAMP, and Syntaxin belong to the class of SNARE (soluble N-ethylmaleimide-sensitive factor attachment protein receptors) proteins, which play an important role in docking of synaptic vesicles with the presynaptic membrane.

The idea behind this review was to see whether BoNT has the ability to act on motor, sensory, and autonomic neural systems other than by producing weakness or inhibiting the neurotransmitters and neuropeptides at the periphery or blocking neurotransmission at the neuromuscular junction alone. Our hypothesis was that a part of the effects on these systems is related to network-level alterations in SMI that occur from chronic injections. An earlier review on the central effects of BoNT gives some suggestions about these indirect central effects [5]. In this review, a preliminary idea of dysfunction in SMI in each of PD, CD, and WC will be presented, followed by a summary of evidence that suggests that BoNT may affect SMI in these conditions.

## 2. Methods

Using relevant Medical Subject Heading (MeSH) keywords “Botulinum toxin” or “BoNT” AND “sensorimotor integration” or “Freezing of gait” or “Tremor” or “Cervical dystonia” or “Parkinson’s disease” or “Writer’s cramp”, PubMed database was searched from inception up to June 2025. Articles in English language and only studies involving human subjects were used as limits in search. The abstracts of all those resultant articles (case reports, case series, randomized trials, observational studies) were then reviewed by both authors separately to look for evidence of central effects of botulinum toxin.

Search results were screened using the following inclusion criteria: (i). The study was either observational or interventional in design, case series, or case report (ii). Study patients were adults (≥18 years) with tremor, freezing of gait (FOG), CD, or WC (iii). Study patients received BoNT as a part of the treatment.

**Figure 2:**
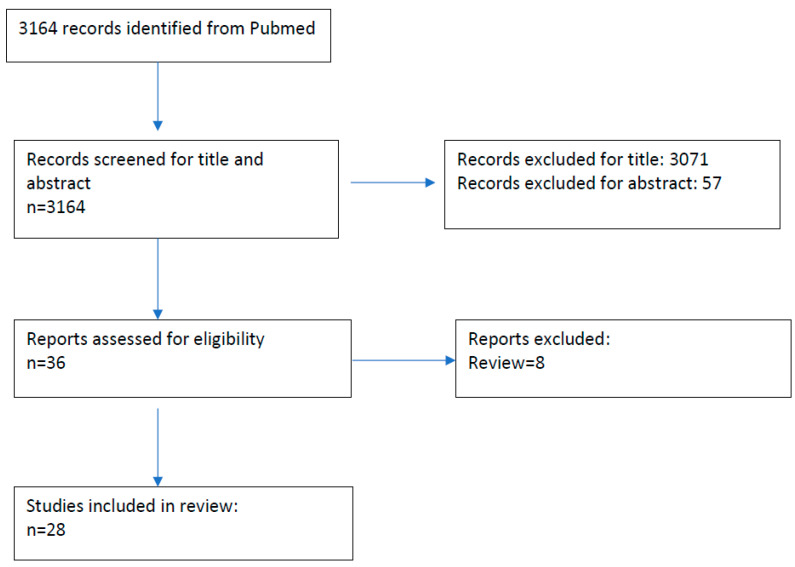
PRISMA flow diagram of the review “Effect of Botulinum toxin on Sensori–Motor Integration in Movement Disorders-A scoping review”.

The relevant articles charted in Excel sheets by both authors for further full-text review, were then matched to see any discrepancy in the number. Any inconsistency was arbitrated by mutual discussion, and, thereafter, the review was performed by summarizing both positive and negative studies of effect of BoNT in SMI. For individual studies, data regarding number of patients, type of study, intervention, and particular conclusions were recorded. This review was not registered. The PRISMA (Preferred Reporting Items for Systematic Reviews and Meta-Analysis) flow diagram for this review is presented in Figure 2, and the PRISMA-ScR checklist has been added as Supplementary Table S1.

## 3. Results and Discussion

### 3.1. A. Parkinson’s Disease (PD)

PD is considered the prototype of aberrant SMI where precision and speed of movement are affected by altered sensory perception [6]. Dopaminergic dysfunction in the pallidum seems to be the reason behind this effect [7]. Recent literature points towards multimodal sensory deficits in PD (proprioceptive, visual, haptic, and auditory perception) manifesting before the onset of motor symptoms and contributing to them [8]. Defects in SMI further impair the central processing of the sensory inputs in the order of hierarchy and relevance. The downstream effect of impaired SMI results in PD patients having impairment of selecting appropriate responses while suppressing inappropriate response tendencies [9]. Also, PD patients have difficulty in suppressing automatic response activation with intact proactive inhibitory control [10,11]. These defects in SMI may manifest clinically as FOG or tremor, which will be discussed below.

FOG is a disabling symptom resulting in falls and injuries. Various hypotheses have been put forward for its pathogenesis, like abnormal gait pattern generation from the central pattern generators of the spinal cord, disruption of the basal ganglia–supplementary motor cortex loop of self-initiated movement, patients becoming increasingly dependent on external clues for compensation via the cerebellum–dorsal premotor cortex and midbrain locomotor region, coupling of abnormal anticipatory postures with initiation of gait, malfunction in perceptual processing of environmental constraints, and associated frontal executive dysfunction [12]. Most of the mechanisms, in a way, center around SMI dysfunction.

There are studies of the use of Botulinum toxin in FOG based on the premise that FOG simulates a dystonic gait [13,14]. Benefits of BoNT in FOG shown in open-label studies [13,14,15] were negated in controlled trials [16,17,18]. A summary of those studies has been compiled in Table 1. A recent systematic review of BoNT in FOG concluded that results from open-label and double-blind studies were conflicting in terms of outcome [19]. However, the underlying hypothesis put forward in some of the studies seems centered around modulation of SMI. When BoNT is injected in the calf and thigh muscles, in addition to producing weakness through its muscle-relaxing properties, it may reorganize muscle activity patterns by altering afferent pathways from the muscle spindles in a local loop [16]. Functional MRI studies in one of the positive studies showed increased activation of cerebellar vermis and nuclei, dorsal pons, and medulla after treatment with BoNT [15]. Central motor patterns may become reorganized after the feedback from the spindles remains altered for the duration of 3 months, or BoNT may directly act on central motor circuits with the inclusion of striatal systems [20,21]. In this way, the afferent pathway or the main SMI circuitry itself may become modulated when BoNT is injected for FOG. However, there is a need for further studies to consolidate this preliminary idea of effect of BoNT in FOG.

Tremor is one of the predominant symptoms of PD, which correlates poorly with other motor or non-motor symptoms. Other features of tremor, such as not progressing at the same rate as bradykinesia/rigidity and responding less to dopaminergic treatment, suggest that tremor might have a different pathophysiology than the classical teaching of dysregulation of direct (underactivity) and indirect (overactivity) pathways being implicated for the motor impairment in Parkinson’s disease [45]. In the finger-dimmer switch model of tremor, tremor is triggered by perturbations in the basal ganglia thalamo–cortical circuits (the finger), which is then transformed into an oscillatory form in the inner circuitry of the thalamus (switch). Finally, the cerebello–thalamic cortical circuit (dimmer) amplifies the tremor and sustains it [46]. The tremor amplitude may subsequently be modulated at the level of the motor cortex [47]. This may be responsible for the rest tremor in Parkinson’s disease. Peripherally, multiple afferent inputs from group 1a muscle spindles occur onto motor neurons when subjects try to maintain a horizontal upper limb posture against gravity, forming a segmental stretch reflex that helps in generation of postural tremor. In Parkinson’s disease, descending exaggerated beta-band oscillations, along with postural drive from the motor cortex, interact with the inputs from muscle spindles to exaggerate this postural tremor [48].

The evidence of action of BoNT in tremor is fairly robust when compared to that in FOG. A systematic review on effect of Botulinum toxin for hand tremor in patients with Parkinson’s disease concluded that it is effective in reducing UPDRS_20 and _21 by 1.22 ± 1.1 and 1.20 ± 0.9, respectively, after 6–16 weeks of injection [49]. In another meta-analysis of randomized controlled trials of hand tremor, BoNT significantly attenuated tremor severity in patients with either essential tremor, Parkinson’s disease, or multiple sclerosis (standardized mean difference = −0.59, 95% confidence interval [CI], −0.95 to −0.24, *p* = 0.001, I2 = 46%) [50]. Efficacy for isolated head tremor has also been proven in multicenter randomized double-blind randomized trial where injections of BoNT into each splenius capitis muscle at day 0 and in week 12 were effective in reducing severity of tremor at 18 weeks [51].

BoNT in tremor acts by weakening the overactive alternate group of muscles (where it is injected) at involved joints. However, few recent papers give us a hint on the effect of BoNT on various parts of SMI, particularly in the context of tremor. The following four studies (Table 1) show how both central integration and peripheral input of SMI can be modulated by BoNT in tremor.

1. Functional MRI studies in patients with multiple sclerosis have shown that reduction in tremor severity following BoNT injection is associated with changes in activation in sensorimotor integration regions like the ipsilateral inferior parietal cortex [22]. The sample size for this study was 43.
2. Following injection, BoNT has been shown to reduce intracortical facilitation and increase long-interval intracortical inhibition, short latency afferent, and long latency afferent inhibition at peak BoNT A time points [23]. This may reduce the effect of the central generator on the tremor of Parkinson’s disease and essential tremor and may be helpful in reducing the oscillations in the cerebello–thalamo–cortical circuit. This study was done in twelve de novo and seven Levodopa-optimized PD patients with tremor affecting one arm.
3. By injecting BoNT around the muscle spindles, which converge at the tendinous ends, it is theoretically possible to block afferent proprioceptive inputs from group 1 muscle spindles forming the reflex arc of the stretch reflex, generating postural tremor/part of kinetic tremor. Something similar to this has been shown in a type of tremor called positional tremor, described by Schaefer et al. [24]. They described two patients with tremor, which occurred when the involved body part was in a particular position during any task, making it posture specific and not task specific. Both of the patients improved after injection of lidocaine in the end plate zone of particular muscle, biceps in one and flexor digitorum superficialis in the other. The authors postulated that abnormal muscle spindle afferent drive from gamma spindle fibers is blocked by injection of liquid lidocaine, thereby improving the tremor. Subsequent injection by botulinum toxin at the same sites confirmed the hypothesis by improving tremor and not causing weakness. However, this mechanism may not hold true in case of rest tremor.

On the same line, efficacy of BoNT for jerky, position-specific, upper limb action tremor was shown in eight patients in a pilot prospective cohort study [25]. Although the authors did not explain the mechanism, a part of the action may be due to proprioceptive input from the muscle spindles becoming modulated following BoNT injection as described in the previous study [24].

So, to summarize, although the evidence for BoNT action in tremor is robust, there is still paucity of data for the fact whether BoNT affects SMI behind its action in tremor. This has also been concluded in one systematic review combining both animal and human data [21]. With only four open-label studies, further studies need to focus specifically on whether BoNT modulates various parts of SMI after chronic injection in tremor by combining neurophysiology, transcranial magnetic stimulation, and imaging data in a larger cohort of patients.

### 3.2. A. Cervical Dystonia (CD)

CD is another disorder where impaired sensory input into the primary sensorimotor cortex disrupts sensorimotor integration. Smeared representations of body parts in primary somatosensory cortex cause generalized somatosensory deficit in cervical dystonia. Both temporal and spatial forms of somatosensory perception, like tactile temporal discrimination threshold, spatial grating sensitivity, and proprioception, are impaired [52].

Due to impaired and overlapping representations of arm/hand and head in the somatosensory cortex, overrepresented (noisy) feedback is provided to the motor cortex. The somatosensory cortex also gives input to the striatum. Due to the impaired gating mechanism in the basal ganglia, Gpi gives a fluctuating inhibitory input to the motor cortex through the thalamus. Subsequent abnormal efferent input from the motor cortex goes to the alpha and gamma motor neurons (innervating the extrafusal muscle fibers and spindles of neck muscles) and cerebellum. The altered proprioceptive input from the spindles goes again to the somatosensory cortex, reinforcing the altered circuitry. It also goes to the cerebellum. The cerebellum consolidates the altered input from the motor cortex and spindles of the neck muscles and, thereafter, gives efferent signals through the thalamus to the motor cortex. All the convergent feedback from the somatosensory cortex, cortico basal ganglia, and cortico–cerebellar loop ultimately leads to an abnormal posture of the head in CD [52].

In addition to the weakening effect of BoNT on the overactive muscles in CD by inhibiting the release of acetylcholine in the synaptic cleft, all of the below-mentioned studies of BoNT (Table 1) hint towards modulation of SMI in CD.

1. Alteration of the proprioceptive input by injecting into the spindles innervated by gamma efferent neurons, along with the muscles (innervated by alpha motor neurons), may potentially reduce the altered input going to the somatosensory cortex and decrease dystonia. This indirect central effect stems from the fact that BoNT restores abnormally increased spatial discrimination thresholds, which is considered a clinical marker of disorganized cortical somatotropy [26].
2. In another study of 15 CD and 15 control participants, Khosravani et al. showed abnormal wrist proprioceptive perception in both symptomatic and non-symptomatic upper limbs during active/passive movements in CD patients, normalized after neck BoNT injections [27]. The authors hypothesized that BoNT injections normalized the cortical processing of proprioceptive information, indicating central function of BoNT.
3. There is also evidence of BoNT normalizing N30 potential amplitudes, which were larger in patients (16 in number) with CD than controls, suggesting its central effects [28].
4. Preliminary neuroimaging studies point at differences between BonT-naïve and BoNT-treated patients with CD with respect to gray matter volume in the precentral sulcus and bilateral mesio-temporal cortices [29].
5. Delnooz et al. did a functional MRI study in 23 CD patients showing that BoNT treatment restored connectivity abnormalities in the sensorimotor and primary visual network [30].
6. In a study of 17 CD patients and 17 controls, baseline increased information flow within the sensori–motor cortex, basal ganglia, and thalamus showed a shift towards normalization in functional MRI at 6 months of BoNT treatment [31].
7. In another study of seven patients and nine healthy controls, Opavsky et al. showed reduced hand movement-related cortical activation but increased blood oxygenation level-dependent signal change in the contralateral secondary somatosensory cortex in patients when compared to controls [32]. Following effective BoNT treatment, sensorimotor maps showed a significant decrease, highlighting the correlation of BoNT effect at the level of central nervous system.
8. With a much higher number of patients (92), Feng et al. showed increased baseline connectivity of right postcentral gyrus with left dorso-medial prefrontal gyrus and right caudate nucleus in patients with cervical dystonia, which was associated with their symptom severity [33]. BoNT reduced this excessive functional connectivity, further establishing central effects of BoNT therapy.
9. Similar functional MRI-based studies have been conducted by Nevrly et al. in 12 patients with CD showing evolution of network level activation as early as 1 month following the first BoNT injection to the dystonic neck muscles [34].
10. An exploratory study of magnetoencephalography with four patients of CD and four controls showed a difference in coherence between controls and patients in the following regions: fronto-striatal, occipito-striatal, parieto-striatal, and striato-temporal networks [35]. Following BoNT injection, there was increased coherence in these areas, which was especially significant in the left putamen and right superior parietal gyrus. The authors concluded that BoNT might affect SMI, which could have an effect on the clinical benefit.
11. Transcranial magnetic stimulation (TMS) based measures of sensorimotor integration, which are mediated through central processes like short latency afferent inhibition (SAI), decreased and finally normalized in patients with cervical dystonia following BoNT injection. This change in SAI correlated with improvement in pain levels. The authors concluded that pain control in CD following BoNT therapy was related to the modulation of SMI [36].
12. A similar TMS-based study on 12 patients with CD showed that paired associative stimulation, which significantly facilitated motor evoked potentials in hand muscles at baseline, did not have a similar effect after 1 month of BoNT injection in the neck muscles. The authors postulated that the modulated afferent input from the neck post-BoNT injection caused reorganization of the motor cortical representation of hand muscles [37].

To summarize, there are a greater number of studies showing SMI modulation by BoNT in CD than that in tremors or FOG. Although most of the studies, except one, are small, they are all positive studies supporting our view.

### 3.3. A. Writer’s Cramp (WC)

The pathophysiology of WC is multifactorial, with a deficient sensory system, abnormal reorganization of the somatosensory cortex, and abnormal sensorimotor integration as a result of maladaptive plasticity [34]. There is also increased excitability and loss of inhibition occurring at multiple levels, like the motor cortex, premotor cortex, somatosensory cortex, and cerebellum [53].

Maladaptive somatotropic finger representations in the primary somatosensory cortex have been established by various neurophysiological studies like defective proprioceptive processing in a tonic vibration task, reduced heat evoked potential and pain rating in quantitative sensory testing, impaired tactile information processing with increased threshold of temporal and spatial discrimination, impaired fine force regulation as in a drawer opening task and increased error, greater variability, and longer release in force tracking tasks [53].

Evidence of WC being a sensorimotor network disorder comes from various imaging studies [53]. Morphometric studies show increased grey matter volume bilaterally in the posterior putamen and globus pallidus, as well as decreased grey matter in the hand area of left sensorimotor cortex, cerebellum, and thalamus. Diffusion weighted analysis and graph theoretical analysis to look for structural connectomes showed reduction of nodes in bilateral putamen, insula, cingulate cortex, cerebellar vermis, left cerebellar lobule VIII, and inferior temporal gyrus [53]. There is a more pronounced BOLD signal in contralateral sensorimotor cortex, supplementary and dorsal premotor cortex, putamen, and thalamus during motor imagery of writing, showing alterations.

Recent studies (Table 1) do give a hint of modulation of SMI when BoNT is used in WC.

1. In one study, treatment with BoNT in focal hand dystonia produced a change in long latency reflexes, reflective of a cortical generator in the supplementary motor area [38].
2. In another study using TMS, the authors demonstrated that injections of BoNT in the affected muscles temporarily reverse the abnormal cortico–motor projections of the hand and forearm muscles in patients with WC [39].
3. A more recent study based on somatosensory evoked potential (SEP) to test sensorimotor integration. However, it did not find any difference between patients and controls at baseline and after BoNT A treatment [40]. It may be argued that SEP may have poor sensitivity in detecting abnormalities in sensory discrimination.
4. Trompetto et al. showed that the tonic vibration reflex was depressed more and for a longer amount of time than the maximal M wave and the maximal voluntary contraction in BoNT-treated patients with WC [41]. They finally postulated that this unique sensitivity of tonic vibration reflex may be due to the chemo denervation of intrafusal muscle fibers, leading to decreased input to the central nervous system and thereby altering sensorimotor integration.
5. In another study on patients with CD and WC, the researchers showed that BoNT reduced abnormal somatosensory temporal discrimination threshold values during movement execution when compared with healthy subjects or patients with blepharospasm [42]. They concluded that BoNT improved abnormal SMI by decreasing the overflow of proprioceptive signaling from muscle dystonic activity to the thalamus.
6. Zeuner et al. showed that BoNT improved force regulation in patients with WC, which is not possible only with muscle weakening and can be explained by better SMI [43].
7. In a PET study on six patients with WC, the authors concluded that although BoNT improved writing, it did not improve the associated dysfunction of primary motor and premotor cortex [44].

The evidence of modulation of SMI by BoNT in WC is mixed. Also, since the number of patients in these studies is small, more studies with reproducible analysis parameters are needed before any relevant conclusion can be drawn. Prospective studies in homogeneous large cohorts receiving multiple treatment sessions of BoNT can provide concrete evidence of its central effects.

## 4. Limitations

Since no critical appraisal and risk of bias assessment of individual studies were done, there is a small potential for a biased conclusion. Also, the review was done by two authors only.

## 5. Conclusions

The evidence for SMI modulation by BoNT is not robust in tremor, FOG, and WC. It is, albeit, better in CD. Most of the evidence, based on small open-label studies and without any long-term follow-ups, gives preliminary support to the fact that, following chronic BoNT injection, altered afferent input from muscle spindles and sensory receptors may influence spinal and cortical circuits with subsequent changes in SMI and brain plasticity. This mechanism may contribute to the beneficial effects of BoNT in PD, CD, and WC, in addition to its overwhelming peripheral effect of causing weakness. Rather than focusing on individual surrogate markers, future studies should combine electrophysiology, imaging, and transcranial magnetic stimulation parameters in larger cohorts of patients with the above-mentioned conditions to further elaborate on the hypothesis of SMI modulation following BoNT injection. As a part of further exploring the possibility of SMI in the use of BoNT, targeting the input systems in muscles such as spindles and other afferents (through electromyography) may be a better way to use BoNT, potentially reducing the amount of toxin injected and the resulting muscle weakness and atrophy.

## Figures and Tables

**Table 1:** Summary of studies showing modulation of sensorimotor integration by Botulinum toxin.

**Freezing of Gait**				
Author with year	Type of study	Number of patients	Intervention	Conclusions
Giladi et al., 2001 [13]	Open-label pilot study	10 patients with Parkinsonism	BoNT	Improvement in 7 out of 10 patients
Giladi et al., 1997 [14]	Case report	1 patient with Parkinson’s disease	BoNT	Improvement of FOG
Vastik et al., 2016 [15]	Cohort study	20 patients with PD, 10 with FOG, and 10 without FOG	BoNT + fMRI	Improvement in FOG patients as in FOG questionnaire scores, fMRI in FOG group showing increased activation in cerebellar vermis and nuclei, dorsal pons, and medulla
Gurevich et al., 2007 [16]	Double-blind placebo-controlled pilot study	11 PD patients, 6 received BoNT and 5 received saline	BoNT/Placebo	No improvement in either group
Wieler et al., 2005 [17]	Double-blind placebo-controlled crossover study	12 patients with PD and FOG	BoNT/Placebo	No significant improvement in subjective and objective measures
Fernandez et al., 2004 [18]	Double-blind, placebo-controlled, parallel-group study	14 patients with PD and FOG, 9 randomized to BoNT and 5 to placebo	BoNT/Placebo	No significant difference between the treatment and placebo arms in the number of patients improved versus unchanged
**Tremor**				
Boonstra et al., 2020 [22]	Randomized controlled trial	43 multiple sclerosis patients with tremor, 21 randomized to BoNT and 22 to placebo	BoNT/Placebo and fMRI	Patients with BoNT-A had improved handwriting tremor at 6 weeks and 12 weeks, and also showed a significant reduction in activation within the inferior parietal lobule
Samotus et al., 2021 [23]	Open-label pilot study	12 medication naïve and 7 levodopa-optimized PD patients with tremor affecting one arm	BoNT and paired pulse transcranial magnetic stimulation	At 6 weeks post treatment, ICF was significantly reduced, while LICI, SAI, and LAI were increased.
Schaefer et al., 2017 [24]	Case series	2 patients with positional tremor	BoNT	BoNT improved positional tremor in 2 patients
Saifee et al., 2016 [25]	Open-label study	8 patients with jerky position-specific upper limb action tremor	BoNT	6 out of 8 patients had improvement in their tremor
**Cervical dystonia**				
Walsh et al., 2007 [26]	Open-label study	20 patients with CD and 18 healthy age-matched controls	BoNT	Improvement in spatial discrimination thresholds by 23% from baseline to 1-month values in the dystonia group
Khosravani et al., 2020 [27]	Open-label study	15 CD and 15 control patients	BoNT	Neck BoNT injections normalized cortical processing of proprioception from asymptomatic limbs in the task of active wrist position mapping
Kanovsky et al., 1998 [28]	Open-label study	16 patients with CD	BoNT	After correction of head movement by BoNT, amplitude of contralateral (to head turn) P22/N30 component was significantly lower than pre-treatment values
Delnooz et al., 2015 [29]	Longitudinal study	23 CD patients and 22 healthy controls	Pre and post-treatment structural MRI	Pre and post BoNT treatment showed increase in gray matter volume in right precentral sulcus. CD patients had also reduced gray matter volume in left ventral premotor cortex
Delnooz et al., 2013 [30]	Longitudinal study	23 CD patients and 22 healthy controls	3 (Pre, at 4 weeks, and before next BoNT) resting state functional MRI analysis	BonT treatment partially restored connectivity abnormalities in sensorimotor and primary visual area
Brodoehl et al., 2019 [31]	Longitudinal study	17 CD patients and 17 healthy controls	Functional MRI at baseline and 6 months of BoNT	Increased connectivity between basal ganglia, thalamus, and sensorimotor cortex, with associated diminished responsiveness to regulating inputs, showed a shift towards normalization following BoNT
Opavsky et al., 2011 [32]	Longitudinal study	7 patients with CD and 9 healthy controls	Functional MRI at baseline during skilled motor task, MRI repeated after 4 weeks of BoNT	BoNT reduced activation of supplementary motor area, dorsal premotor cortex, and basal ganglia
Feng et al., 2021 [33]	Longitudinal study	92 patients with CD and 45 healthy controls	Pre- and post-BoNT injection functional MRI	BoNT-A reduced excessive functional connectivity between the sensorimotor cortex and right superior frontal gyrus.
Nevrly et al., 2018 [34]	Longitudinal study	12 patients with CD (BoNT naïve)	Pre- and post-BoNT injection functional MRI	BoNT treatment was associated with a significant increase of activation in finger movement-induced fMRI activation of several brain areas, especially in the bilateral primary and secondary somatosensory cortex, bilateral superior, inferior parietal lobule, bilateral SMA, and premotor cortex, along with other areas.
Mahajan et al., 2017 [35]	Longitudinal study	4 patients with CD and 4 healthy controls	Pre- and post-BoNT magnetoencephalography	Increased coherence in fronto-striatal, occipito-striatal, parieto-striatal and striato-temporal networks post BoNT therapy in CD patients
Shukla et al., 2023 [36]	Longitudinal study	11 CD patients and 10 healthy controls	Pre- and post-BoNT TMS-based measurements	Increased short latency afferent inhibition in CD patients normalized to healthy control data at the time of peak BoNT effects.
Kojovic et al., 2011 [37]	Longitudinal study	12 CD patients	Pre- and post-BoNT TMS-based measurements	Post-BoNT treatment at 1 month, the effect of paired associative stimulation to facilitate motor evoked potentials in hand muscles significantly reduced.
**Writer’s cramp**				
Naumann et al., 1997 [38]	Longitudinal study	34 with focal hand dystonia and 20 controls. 12 dystonia patients injected with BoNT	Long latency reflexes 1 and 2 after median nerve stimulation	Significant reduction of LLR 2 reflex amplitude on the clinically affected site following BoNT treatment
Byrnes et al., 1998 [39]	Longitudinal study	15 subjects with WC	Pre- and post-BoNT TMS-based measurements	Displaced and distorted corticomotor projection maps of hand and forearm muscles were temporarily reversed during the period of clinical effect of BoNT injection
Contarino et al., 2007 [40]	Longitudinal study	29 WC patients and 10 controls	Median and Ulnar somatosensory evoked potential	No difference between patients and controls in standard SEPs, and also in paired stimulation of median nerve at interstimulus intervals of 40 and 100 ms. No differences persisted after BoNT injection, too
Trompetto et al., 2006 [41]	Longitudinal study	10 right-handed WC patients	Tonic vibration reflex, maximal M wave, and maximal voluntary contraction (MVC) in the injected muscles pre- and post-BoNT	TVR was reduced more than M-max and MVC following BoNT injection
Bartolo et al., 2020 [42]	Longitudinal study	14 CD patients, 11 blepharospasm patients, and 10 patients with focal hand dystonia	Somatosensory temporal discrimination threshold (STDT) at rest and during voluntary movement	BoNT reduced abnormal STDT during movement in CD and FHD patients. No modification of rest STDT
Zeuner et al., 2013 [43]	Longitudinal study	10 patients and 18 controls	Pressing index finger on a force sensor tracking 2 visual targets pre- and post-BoNT	Disturbed fine force control in patients improved after BoNT treatment
Ceballos-Baumann et al., 1997 [44]	Longitudinal study	6 WC patients and 6 healthy controls	H2 15O-PET activation study before and after BoNT	BoNT treatment did not reverse associated dysfunction of primary motor and premotor cortex. An increased activation in parietal cortex and caudal supplementary motor area may represent a change in movement strategy or associated cortical reorganization secondary to deafferentation of alpha motor neurons

## Data Availability

No new data were created or analyzed in this study.

## Supplementary Materials

The following supporting information can be downloaded at: https://www.mdpi.com/article/10.3390/toxins17080416/s1, Table S1: Preferred Reporting Items for Systematic reviews and Meta-Analyses extension for Scoping Reviews (PRISMA-ScR) Checklist.

## Abbreviations

The following abbreviations were used in this manuscript:

BoNT	Botulinum toxin
PD	Parkinson’s disease
CD	Cervical dystonia
WC	Writer’s cramp
SMI	Sensori–motor integration
SMA	Supplementary motor cortex
SNAP 25	Synaptosomal-associated protein
VAMP	Vesicular-associated membrane protein
FOG	Freezing of gait
TMS	Transcranial magnetic stimulation
SAI	Short-latency afferent inhibition
